# How much precision in reporting statistics is enough?

**DOI:** 10.3325/cmj.2015.56.490

**Published:** 2015-10

**Authors:** Farrokh Habibzadeh, Parham Habibzadeh

**Affiliations:** 1 Shiraz University of Medical Sciences, Shiraz, Iran; 2 Medical Education and Research Center, Petroleum Industry Health Organization, Shiraz, Iran; 3 Student Research Committee, Shiraz University of Medical Sciences, Shiraz, Iran

## How much precision in reporting statistics is enough?

Depending on the accuracy of the tools we employ in our research, each variable is measured within a certain degree of precision. For example, in most clinical studies on adults, age is measured in years. Generally, measuring the age with more accuracy in such studies is neither necessary nor of any particular importance. However, we might measure blood pH in the same study with two or even three digits after the decimal point because minute changes in blood pH are associated with serious clinical implications. Statistical software programs commonly used in the analysis of research data, however, calculate the results with a predefined precision, say, three digits after the decimal point, no matter how accurately the raw data were measured. Therefore, the software would report the mean of both of the mentioned variables, age and pH, with three digits after the decimal point.

The question arises: how should we report these statistics in scientific articles? Apparently, there is no consensus on this issue. For example, some references suggest that in reporting statistics (eg, means and standard deviations [SDs]) not to use precisions higher than the accuracy of the measured data (1); many researchers recommend to use only one decimal place more than the precision used to measure the variable (2,3); and, some mention that although means should not be reported to no more than one decimal place more than that of the raw data, SDs may need to be reported with an extra decimal place (4). Considering the existing controversy and the importance of this issue, in this commentary, we try to provide a reasonable answer to this question.

Suppose that variable *x *is measured with precision of* α *and reported as 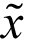. Then, we can write:


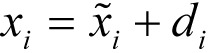
_(Eq. 1)_

where,


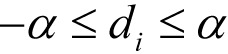
_(Eq. 2)_

Then,


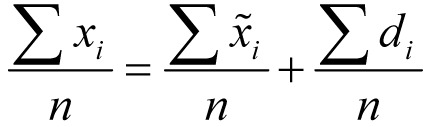


which yields,


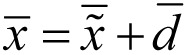


and,


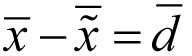


But, considering Eq. 2, we have:


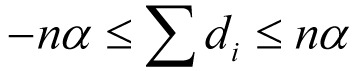


and


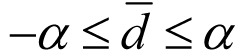


Thus,


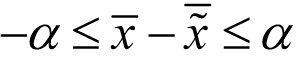


which means that the precision of measurement of the mean, 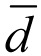, is typically expected to be near zero because errors in the measurements are presumably random – some are positive and some are negative. However, the absolute error in general would be ≤α. Therefore, the mean value cannot be reported with a precision higher than that used in the measurement of the raw data.

For the variance (SD^2^), beginning with Eq. 1, we will have:


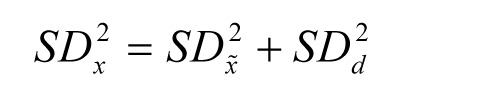
_(Eq. 3)_

But,


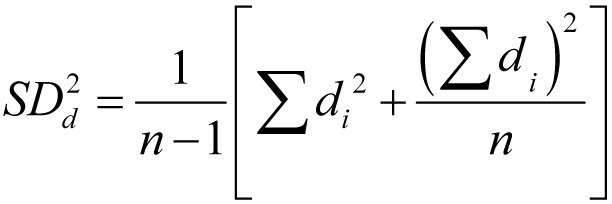


The second term in the squared bracket is negligible and the above equation then becomes:


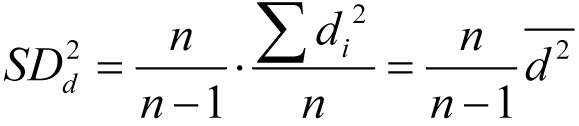


Combining this equation with Eq. 3, yields:


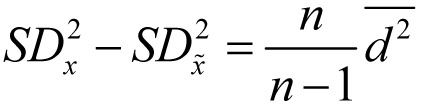


Then,


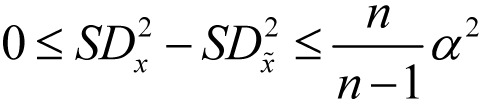


The most probable error in the calculation of the variance (SD^2^) would be:


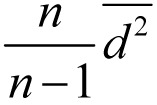


But, theoretically it can be as high as


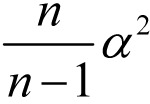


This value is 2α^2^ when the sample size is only two; for large sample sizes, however, it would be almost α^2^. Considering the amount of variability in the variance (SD^2^), the precision of the SD is therefore no more than α. As a result, it seems reasonable to conclude that SDs should also not be reported with a precision more than the accuracy of the measured raw data.

## Example

Suppose that we measured serum total bilirubin of 10 newborn patients with hyperbilirubinemia. In a clinical laboratory, total bilirubin is normally measured with a precision of ±0.05 mg/dL (one digit after the decimal point). For example, all values of total bilirubin between 5.35 and 5.44 mg/dL would be recorded as 5.4 mg/dL. Assuming the second column of Table 1 (measured values) is our readings, columns 3 to 5 would be possible more accurate values for the measured bilirubin levels. Considering the accuracy in the measurement of total bilirubin in a clinical laboratory (±0.05 mg/dL), all these data sets (Table 1: columns 2 to 5) are practically identical (to one digit after the decimal point).

**Table 1:** Serum total bilirubin of 10 newborns measured with a precision of ±0.05 mg/dL

	Measured values	Three possible values		
3.2	3.22	3.23	3.20
3.1	3.14	3.14	3.13
4.5	4.45	4.51	4.47
8.2	8.23	8.21	8.19
9.3	9.34	9.27	9.28
11.7	11.73	11.65	11.65
10.0	10.01	10.02	9.99
10.8	10.84	10.79	10.78
7.1	7.14	7.13	7.09
6.8	6.84	6.82	6.82
**Mean (standard deviation) reported with ±0.05 mg/dL (One digit after the decimal point)**	7.5 (3.1)	7.5 (3.1)	7.5 (3.1)	7.5 (3.1)
**Mean (standard deviation) with reported ±0.005 mg/dL (Two digits after the decimal point)**	7.47 (3.09)	7.49 (3.10)	7.48 (3.07)	7.46 (3.08)

Because our measurement precision was ±0.05 mg/dL (one digit after the decimal point), according to what we found above, the precision to be used for reporting mean and SD should also be ±0.05 mg/dL (one digit after the decimal point). The means and SDs reported for all these practically similar data sets are not different if they are reported with the same accuracy we used to measure the raw data (7.5 [SD 3.1] mg/dL). However, if we report the mean and SD with more precision than the accuracy we used to measure the raw data (eg, two digits after the decimal point) we have a mean of 7.47 (SD 3.09) mg/dL, which is different for the real means and SDs of other possible data sets (Table 1).

The precision for reporting of each statistic depends on how that statistic is derived. As an example, if the precision of a measurement is ±α (say ±0.05, one digit after the decimal point), while we should report mean and SD with the same precision, we need to report the variance (SD^2^) with two digits after the decimal point (α^2^ = 0.0025, assuming a large sample size). Because in the calculation of all percentiles (including 25th, 50th [median], and 75th percentiles) we use linear calculations, all percentiles (including the interquartile range [IQR]) should be reported with a precision not higher than the measurement precision (like reporting mean and SD).

Reporting statistics with more than necessary precisions would be misleading (5,6). The number of decimal places to be reported for the mean, SD, median, and IQR in scientific reports should not exceed that of the precision of the measurement in the raw data.
